# Blue to yellow emission from (Ga,In)/GaN quantum wells grown on pixelated silicon substrate

**DOI:** 10.1038/s41598-020-76031-3

**Published:** 2020-11-03

**Authors:** Benjamin Damilano, Marc Portail, Eric Frayssinet, Virginie Brändli, Florian Faure, Christophe Largeron, David Cooper, Guy Feuillet, Daniel Turover

**Affiliations:** 1 Université Côte D’Azur, CNRS, CRHEA, Rue B. Gregory, Valbonne, France; 2 Univ. Grenoble Alpes, CEA, LETI, 38000 Grenoble, France; 3 SILSEF, 382 Rue Louis Roustin, 74160 Archamps, France

**Keywords:** Lasers, LEDs and light sources, Semiconductors, Materials for optics

## Abstract

It is shown that substrate pixelisation before epitaxial growth can significantly impact the emission color of semiconductor heterostructures. The wavelength emission from In_x_Ga_1−x_N/GaN quantum wells can be shifted from blue to yellow simply by reducing the mesa size from 90 × 90 µm^2^ to 10 × 10 µm^2^ of the patterned silicon used as the substrate. This color shift is mainly attributed to an increase of the quantum well thickness when the mesa size decreases. The color is also affected, in a lesser extent, by the trench width between the mesas. Cathodoluminescence hyperspectral imaging is used to map the wavelength emission of the In_x_Ga_1−x_N/GaN quantum wells. Whatever the mesa size is, the wavelength emission is red-shifted at the mesa edges due to a larger quantum well thickness and In composition.

## Introduction

Group-III nitride semiconductors are the materials of choice for the fabrication of blue and white light emitting diodes^1^. The active region is made of blue light emitting (Ga,In)N quantum wells (QWs) heterostructures encapsulated in a yellow/red emitting luminophore which converts part of these blue photons to generate white light. These devices are now the most efficient white light sources for general lighting^2^. However, the color of these lamps can only be weakly tuned. Fine control of the light emission over the visible range is only possible if colors can be controlled separately. Ideally, an independent control of blue, green and red colors opens up a wide color range. In principle, this can be done easily by mixing three different LEDs: for example (Ga,In)N-based for blue and green, and (Al,Ga,In)P for red. However, with the aim of developing high resolution and high luminance micro-displays^3,4^, this approach is no longer possible. Mixing the three basic blue/green/red colors directly on the same wafer is therefore highly desirable and constitutes an important challenge. Efforts towards this objective have been reported in the literature using for example nanowires grown by molecular beam epitaxy^5,6^, light conversion by (Ga,In)N multiple quantum wells^7^, facets with different orientations^8^, and local etching of red-emitting LED structures^9^.

Here, we propose the use of mesa-patterned silicon substrates to tune the light emission of (Ga,In)N quantum wells. Mesa-patterned silicon substrates are known to be an efficient way to avoid crack formation in thick GaN structures^10–13^. This is achieved by stress relaxation at the mesa edges that compensate the tensile strain in the GaN layers grown on silicon arising from the thermal expansion coefficient mismatch between GaN and silicon^10–13^. Mesa-patterned silicon substrates have been successfully exploited for the fabrication of devices such as efficient visible LEDs on silicon substrate^14^, laser diodes^15^, exciton–polariton laser^16^, LEDs incorporating a high reflectivity distributed light reflector^17^, high electron mobility transistors^18^. Xu et al. reported an enhanced indium incorporation in (Ga,In)N QWs at the corner of 340 × 340 µm^2^ mesas grown on patterned silicon substrate^19^ but mesa-patterned silicon substrates have never been studied with the objective of modifying the emission color as a function of the mesa size.

## Results

**Figure 1:**
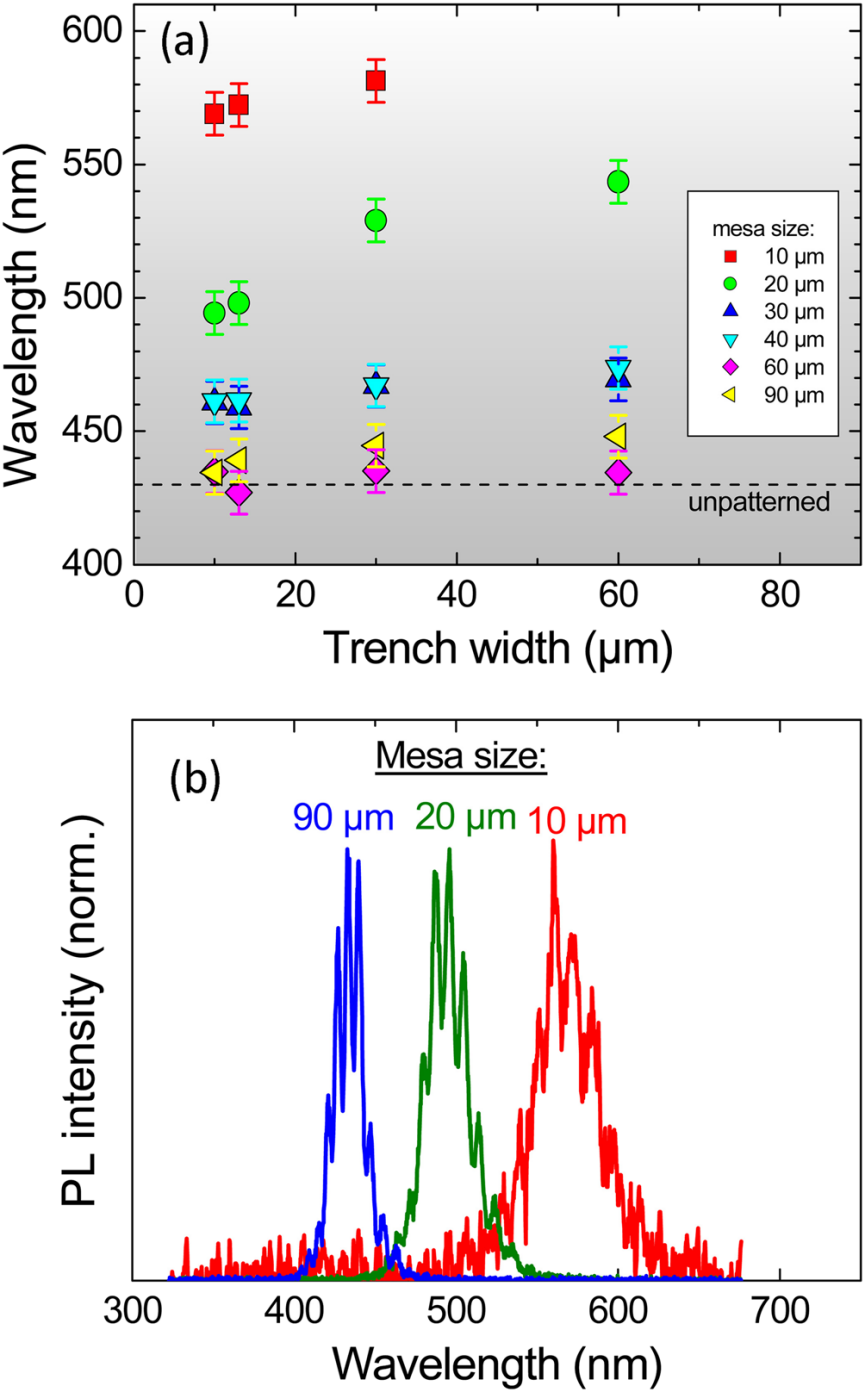
(**a**) Room temperature micro-photoluminescence peak wavelength from the InGaN/GaN multiple quantum well at the center of the mesas with variable sizes and trench widths. The dashed line corresponds to the emission at the center of the reference sample without patterns. (**b**) Examples of spectra with normalized intensities obtained for variable mesa sizes L = 10, 20, and 90 µm with a constant trench width of 10 µm (mesas L10W10, L20W10, and L90W10).

In order to determine the impact of substrate patterning, the micro-photoluminescence was measured at room temperature at the center of 24 different mesas. The sample is divided into different regions with a surface area of 4 × 6 mm^2^. Each region contains a square array of square mesas, each with a side length L and a trench width W. There are 6 different mesas sizes: L = 10, 20, 30, 40, 60, 90 µm and 4 different trench widths W = 10, 13, 30, 60 µm, totalizing 24 different regions. In the following the mesas are identified by LxWy, where x is the side length and y the trench width in µms. The peak wavelength of the (Ga,In)N/GaN MQW extracted from a Gaussian fit of the PL spectra is shown in Fig. 1a. Some of the spectra are given as an example in Fig. 1b for different mesa sizes and a trench width of 10 µm. Note that the PL spectra are structured by oscillations coming from the interference of light in the cavity formed by the nitride layers sandwiched by the air and the silicon substrate. The main result shown by the Fig. 1a is that the PL wavelength increases from ~ 430 to 440 nm for the largest mesa sizes of 60–90 µm to ~ 570–580 nm for the smallest mesa size of 10 µm. This shows that simply by structuring the substrate, different color emissions (at least from blue to green and yellow) can be monolithically integrated on the same sample in one single growth run. The wavelength emission also increases when the trench width increases however to a lesser extent than for the dependence observed as a function of the mesa size. This variation is the largest for the mesas with side length L = 20 µm (Fig. 1a): the peak PL wavelength shifts from 494 nm (blue-green) to 544 nm (green) when varying the trench width from 10 to 60 µm. Some variation is found in the data, for example the QW emission of L60W60 is at slightly shorter wavelength than the one of L90W60. This is due to the non-perfect homogeneity of the QW on the 2-inch wafer during the growth (see Supplementary Information, Figure S1).

The integrated photoluminescence intensity from the center of the mesas as a function of the peak wavelength emission follows an asymmetric bell shape curve. It is maximum in the 470–500 nm wavelength range and decreases by a factor 2.5 at 430 nm and by a factor 100 at 580 nm. The PL signal of the mesa L10W60 is too weak for a reliable estimation of the peak wavelength emission.

**Figure 2:**
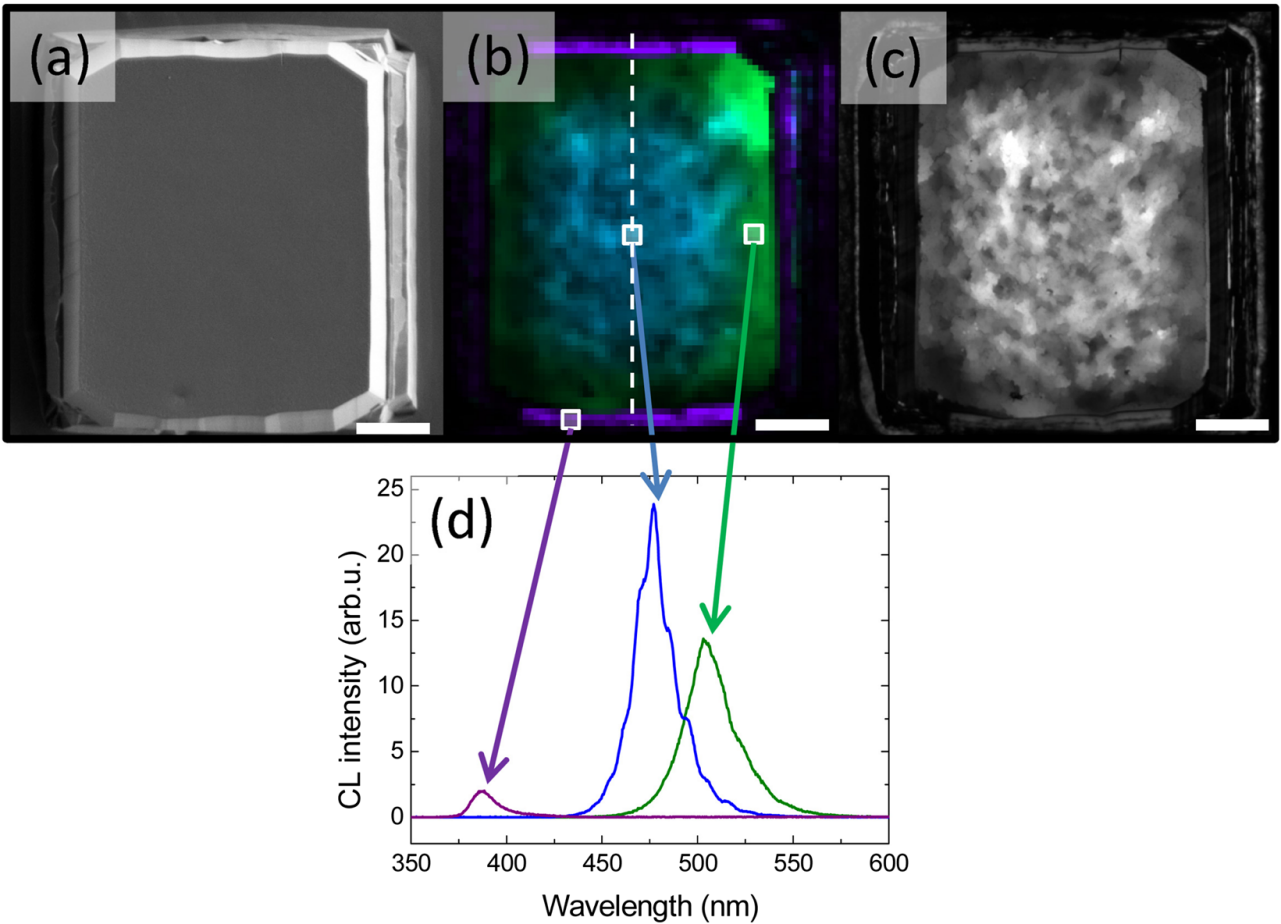
(**a**) Scanning electron microscopy image of a mesa with a side length of L = 40 µm and a trench width W = 30 µm. (**b**) Cathodoluminescence map of the same mesa. The regions corresponding to a wavelength of 390, 475, and 510 nm (spectral width of 10 nm) are highlighted in violet, blue, and green, respectively. (**c**) Corresponding cathodoluminescence panchromatic image. (**d**) Characteristic examples of the spectra obtained in three different regions of the mesa: center, edge, and side facet. The white scale bar in (**a**), (**b**) and (**c**) is 10 µm.

**Figure 3:**
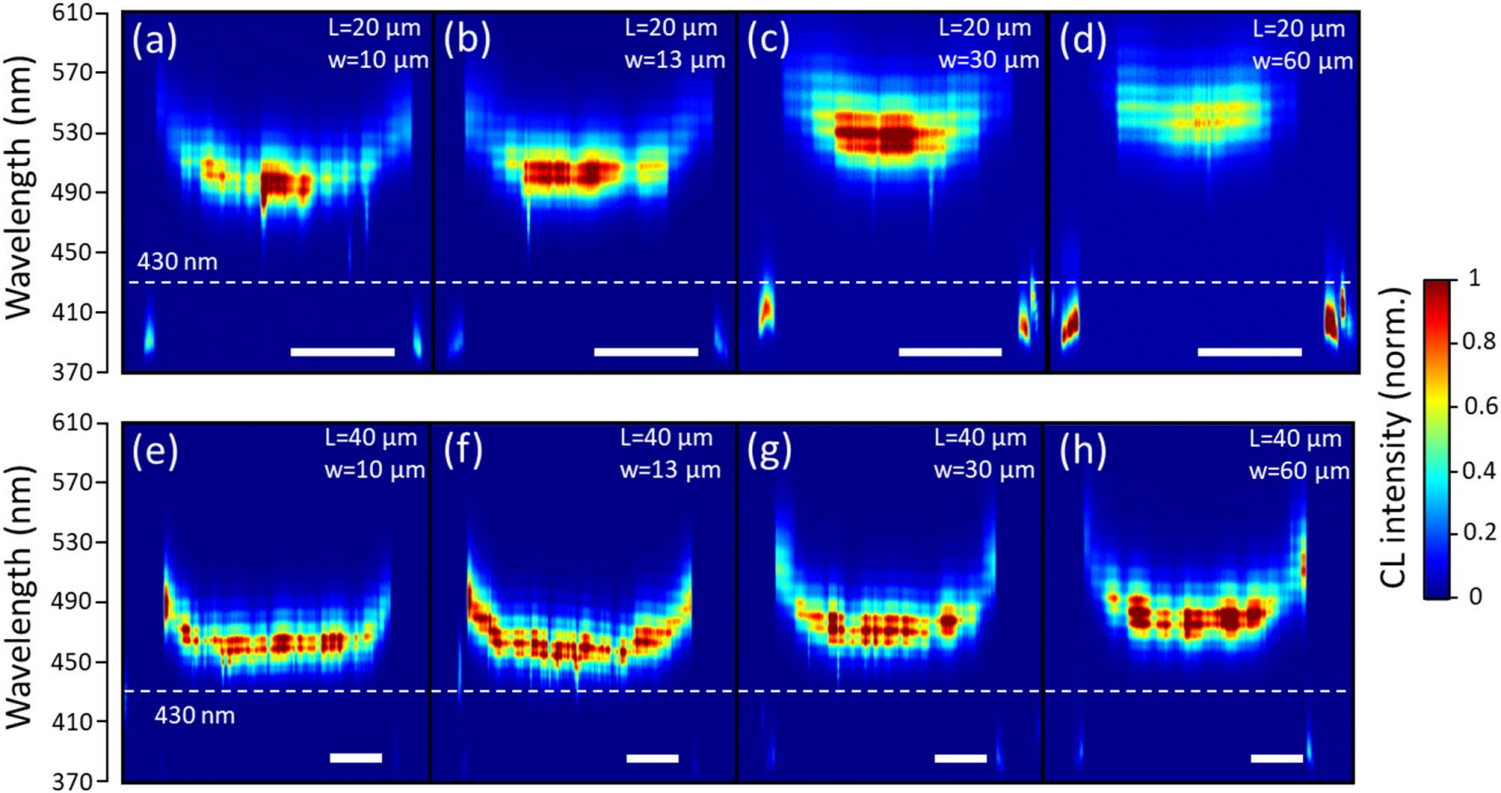
Room temperature cathodoluminescence spectra along a line in the middle of the mesas with a side length L = 20 µm (**a**–**d**) or L = 40 µm(**e**–**h**).The width in between mesas is W = 10 µm (**a**,**e**), 13 µm (**b**,**f**), 30 µm (**c**,**g**), 60 µm (**d**,**h**). The intensity (normalized) of the spectra is color coded from blue (0) to red (1). The doted white line corresponds to a wavelength of 430 nm,as obtained for the unpatterned substrate. The white scale bar is 10 µm in all figures.

Cathodoluminescence (CL) mapping of the mesas was used to obtain spatially resolved spectra from each mesa type, with a step size of 700 nm. One example is shown in Fig. 2, where a mesa L40W30 is investigated (scanning electron microcopy (SEM) image in Fig. 2a). Figure 2b shows the CL intensity map for 3 different wavelengths: 390 nm, 475 nm and 520 nm (corresponding to the violet, blue and green colors). The spectra recorded at 3 different positions on the mesas are shown in Fig. 2d. Blue CL emission is observed at the center of the mesa while the color is shifting towards the green at the edge of the mesa. Violet emission is observed on the inclined facets located at the edges of the GaN mesa. The CL spectra extracted at the center of the mesa following the white dotted line in Fig. 2b are shown in Fig. 3g. This profile indicates that the PL emission is rather homogeneous at the center of the mesa and shifts to longer wavelength at ~ 10 µm from the mesa edge. The panchromatic image of Fig. 2c indicates a slightly weaker intensity at the edges of the GaN mesa. This decrease in the internal quantum efficiency is the typical evolution expected from In_x_Ga_1−x_N/GaN MQWs when the wavelength increases^20,21^.

The CL mapping experiments have been repeated on different mesas and in order to reduce the time required for these measurements, only line scan analysis at the center of the mesas were performed. The results are shown in Fig. 3a–h for mesas L20W10, L20W13, L20W30, L20W60, L40W10, L40W13, L40W30, and L40W60. As reported above for µPL experiments, the peak CL wavelength at the mesa center shifts towards longer wavelength when the mesa size decreases and the trench width between mesas increases. For all the mesas investigated, the peak CL wavelength at the mesa edges is red-shifted compared to the value at the center of the mesa.

## Discussion

The origin of the large emission red-shift when the mesa size decreases and the trench width increases can be explained. Firstly, the fundamental energy of the e_1_-hh_1_ excitonic transition of the In_x_Ga_1−x_N/GaN quantum well is given by the following expression^22^:

1 $$ E_{QW} (x,h) = E_{g} (x) + e_{1} (x,h) + hh_{1} (x,h) - Ry(x,h) - eF(x)h, $$

where E_g_ is the bandgap energy of In_x_Ga_1−x_N, e_1_ and hh_1_ are the confinement energies of the electron and hole, Ry is the exciton binding energy, F is the internal electric field, e is the electron charge, and h is the quantum well thickness.

A red shift of the emission could be due to an increased Indium composition. Indeed, as the In composition x increases the emission energy of the QW decreases because the bandgap of In_x_Ga_1−x_N narrows and in addition the internal electric field increases. Similarly, an increase of the QW thickness can cause a decrease of E_QW_, mainly due to the term –eFh in (1). The strain state of the In_x_Ga_1−x_N QWs can cause a change in the bandgap energy E_g_ and also of the internal piezoelectric field F. Indeed, strain relaxation at the mesa edges can cause a PL blue-shift of the In_x_Ga_1−x_N QWs due to a reduction of the internal piezoelectric field. This last effect has been shown to have a significant impact for very small nanostructures: the PL emission of red-emitting In_x_Ga_1−x_N QWs is shifted to green and blue when they are selectively etched to form nanowires with diameters of 150 and 50 nm, respectively^9^. In our work, the minimum mesa size is 10 µm and therefore a strong impact of strain variation on the In_x_Ga_1−x_N bandgap and the piezoelectric field at the center of the mesas is not expected.

As described above and shown in Fig. 1b, the PL spectra are structured by interference fringes. The spacing of these fringes is characteristic of the total nitride layer thickness grown on the silicon substrate^23^. At a wavelength around 460 nm, at the mesa center, this spacing is estimated to be 8.3 nm and 6.3 nm for the mesas L90W10 and L20W10, respectively. This clearly shows that the total thickness of the nitride layers is larger for L20W10 compared to L90W10.

**Figure 4:**
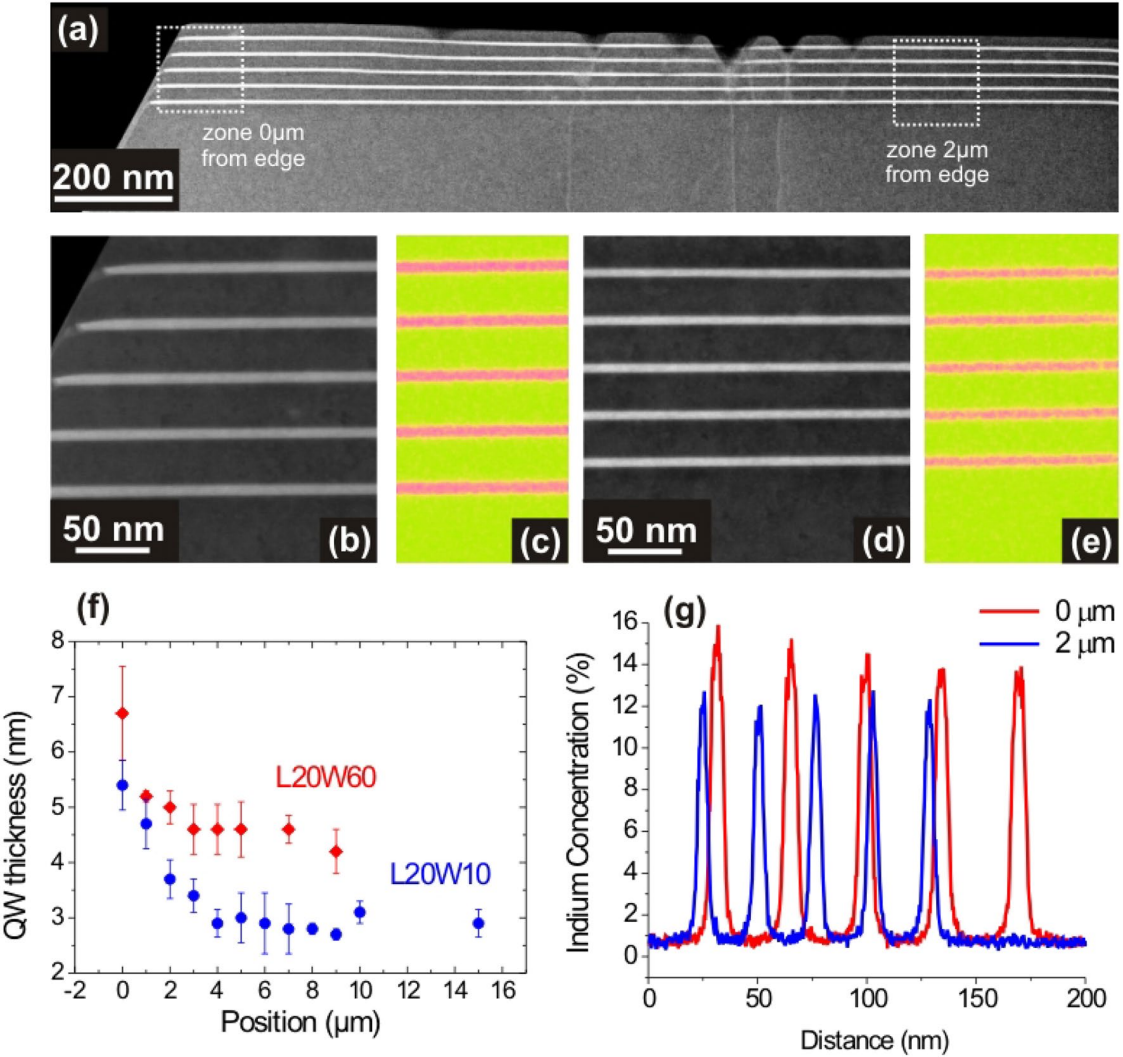
(**a**) HAADF STEM cross section images taken in the top area of a mesa L20W60 (side length of 20 µm and trench width of 60 µm). (**b**) shows a higher magnification STEM image at the mesa edge and (**c**) an EDX map showing the InGaN layers. (**d**) A higher magnification STEM image acquired 2 µm away from the mesa edge and (**e**) an EDX map showing the InGaN layers. (**f**) The InGaN quantum well thicknesses extracted from the transmission electron microscopy images as a function of the position (0 corresponds to the mesa edge) for mesa with a side length of 20 µm and a trench width of 60 µm (in red) or 10 µm (in blue). (**g**) Quantitative profiles of the Indium content extracted from the EDX maps both at the edge and 2 µm away from the edge for the mesa L20W60.

In order to obtain direct and more quantitative data, TEM experiments were conducted to measure the In_x_Ga_1−x_N QW thicknesses. Figure 4a shows cross section HAADF STEM images at the top of a mesa with a side length of 20 µm and a trench width of 60 µm (L20W60) showing the In_x_Ga_1−x_N quantum wells at different positions. Figure 4b shows a higher magnification HAADF STEM image at the mesa edge and Fig. 4c shows a corresponding EDX map. Figure 4d shows a HAADF STEM image acquired 2 µm away from the mesa edge and Fig. 4e the corresponding EDX map. These experiments were repeated at other positions of the mesa L20W60 and also for mesa L20W10. The QW thicknesses were extracted from all these images and are shown in Fig. 4f as a function of the distance from the mesa edge. The In_x_Ga_1−x_N QW thickness tends to a constant at the center of the mesa of about 2.9 nm and 4.5 nm, for L20W10 and L20W60, respectively. Other thickness measurements were performed by TEM on a mesa L10W10. The In_x_Ga_1−x_N QW thickness at the center of this mesa is 5 nm (Fig. S2 of Supplementary Information). These values are significantly larger than the nominal In_x_Ga_1−x_N QW thickness of 2 nm (on a planar substrate). Calculations of the e_1_-hh_1_ fundamental transition of the In_x_Ga_1−x_N/GaN quantum well were performed to determine whether the thickness variation can account for the wavelength shift at the center of the mesas. This calculation was performed using the nominal indium content of 0.13 and the other parameters (bandgap, piezoelectric constants,…) were taken from Ref.^24^ and summarized in Table S1 of Supplementary Information. The calculated QW emission wavelengths are 430, 460, 525, and 551 nm for QW thicknesses of 2, 2.9, 4.5, and 5 nm, respectively. Therefore, this QW thickness increase can account for a large part the observed red-shift of the QW emission energy at the mesa center when the mesa size decreases.

EDX measurements were performed at the edge of mesas L20W10 and L10W10 and give an average In composition of 13.8 ± 2.0% and 18.6 ± 2.0%, respectively. This increase of the In composition can also explain the red-shift of the photoluminescence emission of the mesas when their size decreases. The exact quantification of these EDX measurements in such small QW structures is complicated. However, all the observations were made using identical experimental conditions and quantification, such as the relative values can be compared. The difference in In concentration is also confirmed by the increase in intensity measured by HAADF in the InGaN wells.

The HAADF STEM images indicate a strong increase of the QW thicknesses above a few micrometers from the mesa edges. This is again in good agreement with the wavelength redshifts observed close to the mesa edges for all the CL spectra shown in Fig. 3. More specifically for the mesas L20W10 and L20W60, the CL peak wavelength of the In_x_Ga_1−x_N/GaN MQW also starts to shift at a few micrometers from the mesa edge as shown in Fig. 3a,d. To evaluate the variation of the indium composition of the In_x_Ga_1−x_N QWs the EDX measurements that were performed on the mesa L20W60 are shown in Fig. 4g. The quantitative In composition measurements show an average value of 13.8 ± 2.0% at the edge and 11.6 ± 2.0% at a position of 2 µm from the edge. Indeed, this composition increase also contributes to the wavelength emission red-shift of the In_x_Ga_1−x_N /GaN MQW at the mesa edges.

The enhanced growth at the edges is similar to the observations made by Honda et al.^12^ and attributed to the mass transport of chemical species from the SiO_2_ mask to the mesa edges. Honda et al. observed a strong GaN thickness variation between the center and the edge of the mesas for a mesa spacing larger than 50 µm, while it decreases down to 10% when the spacing between mesas is 10 µm and the mesa size 200 µm. However, in our case, growth occurs in the trenches between the mesas and the growth enhancement should be attributed to another effect such as a perturbation of the critical layer in the gas phase close to the mesa edges^25^. The slightly larger In incorporation at the mesa edges can be related to a stress relaxation effect by the free edges. Indeed, the In_x_Ga_1−x_N layer grown close to the mesa edge can be submitted to a lower compressive stress compared to the one at the center of the mesa and this reduction of stress can induce a larger In incorporation as shown for In_x_Ga_1−x_N quantum wells grown on relaxed (Ga,In)N layers^26,27^.

## Conclusion

We have shown that the wavelength emission from an In_x_Ga_1−x_N/GaN MQW grown on a micro-pixelated Si substrate can be tuned from blue (430 nm) to yellow (580 nm) depending on the pixel size and the trench width between the pixels. Decreasing the pixel size and increasing the trench width leads to a longer wavelength emission for mesas with a side length smaller than 40 µm. This effect is mainly due to an increase of the QW thickness as the mesa side length decreases. The emission is relatively homogeneous at the center of the mesa but a red shift is observed near the mesa edges, again due to an increase of the QW thickness. The efficiency of the yellow-emitting mesas is much smaller than for blue and green. Further optimization is required to get blue, green and red colors on the same wafer. The mesa sizes, pitches have to be finely tuned as a function of QW thickness and indium composition. This approach should then become promising for the fabrication of monolithic full-color micro-display emitters.

## Methods

### Patterned silicon substrate fabrication

The 2-inch Si(111) substrates were covered with a hard mask made of a tri-layer SiO_2_-Al-SiO_2_ and subsequently patterned by direct laser photolithography using an Heidelberg MGP 1 machine. The Si mesas were etched using fluorine (SF6) based inductively coupled plasma (ICP) to provide high aspect ratio silicon etching.

**Figure 5:**
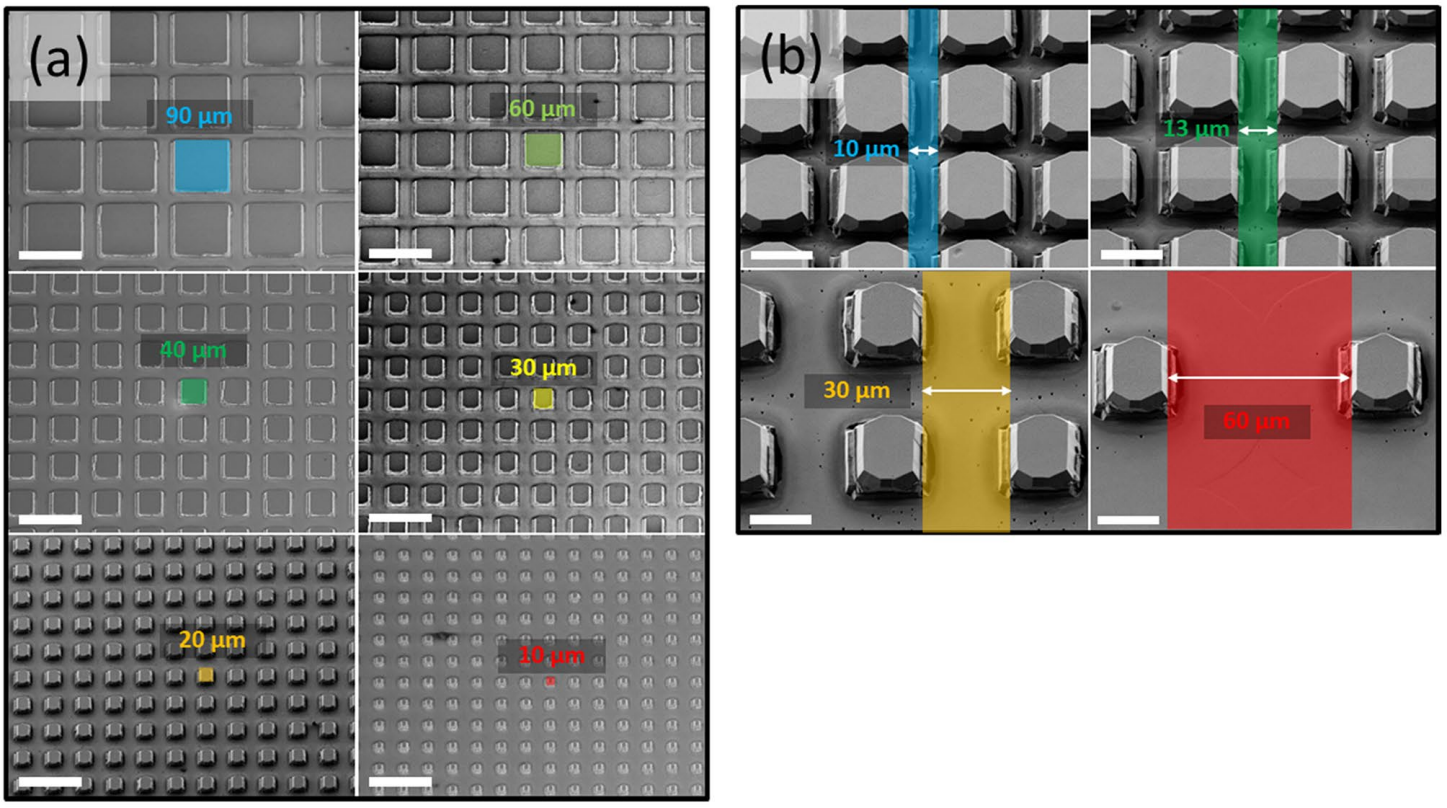
Scanning electron microscopy images at a tilted view of 30° of mesas with variable sizes L = 10, 20 30, 40, and 60 µm and a constant trench width W = 30 µm (**a**) and mesas with a constant size L = 20 µm and variable trench widths W = 10, 13, 30, and 60 µm (**b**). The scale bar is respectively 100 and 20 µm for (**a**) and (**b**).

The sample is divided into different regions with a surface area of 4 × 6 mm^2^. Each region contains a square array of square mesas, each with a side length L and a trench width W. There are 6 different mesas sizes: L = 10, 20, 30, 40, 60, 90 µm and 4 different trench widths W = 10, 13, 30, 60 µm, totalizing 24 different regions. The trench depth in the silicon substrate is 10 µm. SEM images of some of the mesas obtained after growth are shown in Fig. 5.

### Epitaxial growth

The samples were grown on the patterned Si(111) substrates as described above using low-pressure metal organic vapor phase epitaxy (MOVPE) in a commercial close coupled 7 × 2-in. showerhead reactor. The growth rates of the different layers are monitored in situ by a laser reflectivity set-up. Trimethylaluminium, trimethylgallium (for GaN growth), triethylgallium (for InGaN growth) and trimethylindium are used as group-III precursors while ammonia is used as group-V precursor. The structure of the samples comprises (starting from the substrate) a 220-nm thick AlN buffer layer, a 2 µm-thick GaN layer and a 5 periods In_0.13_Ga_0.87_ N (2 nm)/GaN (12 nm) multiple quantum well (MQW). More details about the nucleation procedure on the silicon substrate can be found in Ref.^28^. The first stage of the GaN layer is grown in a 3-dimensional growth mode to reduce the threading dislocation density. On a separate sample grown without the MQW on patterned silicon, a threading dislocation density of 1–2 × 10^9^ cm^−2^ was found emerging at the surface as measured by atomic force microscopy. The room temperature photoluminescence of such QW samples on un-patterned silicon (this sample was grown in the same growth run than the sample on patterned silicon substrate) is centred at 430 nm.

### Characterization

The surface of the samples was imaged by scanning electron microscopy. The micro-photoluminescence (µPL) was measured at room temperature using the 244 nm line of a frequency doubled Argon laser with a power density of 10 W/cm^2^. The laser spot diameter is ~ 2 µm. Cathodoluminescence (CL) was also performed at room temperature both in panchromatic and spatially and spectrally resolved modes.

High resolution scanning transmission electron microscopy (STEM) was performed using a probe corrected FEI Themis operated at 200 kV. Thin specimens were prepared using focused ion beam (FIB) milling in a FEI Strata 400 dual beam operated at 16 kV to reduce specimen damage. The specimens were then cleaned using 2 kV ions. On the different specimens, high-angle annular dark field (HAADF) STEM was performed on the same mesa structures looking down both the 0120 and 0110 zone axis. The indium content in the specimens was evaluated by comparing the relative intensity of the HAADF signal which is sensitive to the Z number in the specimens. In addition, energy dispersive X-ray spectroscopy (EDX) was performed in order to retrieve the relative indium concentrations. Quantification of the In concentration was performed using the Cliff-Lorimer method^29^. Although it is difficult to quantify at these small dimensions using EDX, we assume that the relative concentrations in the same specimens that are measured using the same TEM settings, can be interpreted qualitatively.

## Supplementary information

Supplementary Information
