# Supplementary material for: Blue to yellow emission from (Ga,In)/GaN quantum wells grown on pixelated silicon substrate
Source: Sci Rep. 2020 Nov 3;10:18919. doi: 10.1038/s41598-020-76031-3 (PMC7641114; doi:10.1038/s41598-020-76031-3)
Supplement: Supplementary file 1 — Supplementary Information [file 41598_2020_76031_MOESM1_ESM.docx]

**Supplementary information for**

**“Blue to yellow emission from (Ga,In)/GaN quantum wells grown on pixelated silicon substrate“**

Benjamin Damilano^1,^^[[1]](#footnote-1)^, Marc Portail^1^, Eric Frayssinet^1^, Virginie Brändli^1^, Florian Faure^2^, Christophe Largeron^2^, David Cooper^2^, Guy Feuillet^2^, Daniel Turover^3^

*^1^Université Côte d'Azur, CNRS, CRHEA, Rue B. Gregory, Valbonne, France*

*^2^* *Univ. Grenoble Alpes, CEA, LETI, F-38000 Grenoble, France*

*^3^SILSEF, 382 Rue Louis Roustin, 74160 Archamps, France*


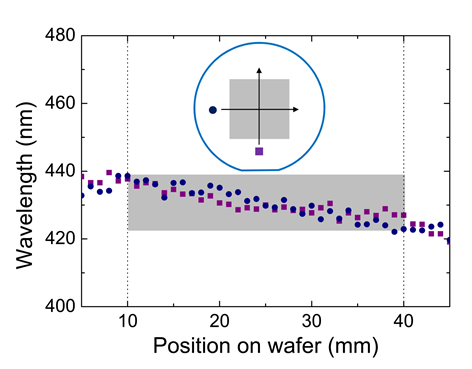


*Figure S1. Peak photoluminescence wavelength of the emission of an InGaN/GaN multiple quantum well grown on an un-patterned Silicon substrate as a function of the position on the wafer as illustrated in the schematics. This sample was grown in same run than the pixelated silicon substrate extensively studied in this work. The emission wavelength at the center of the wafer is 430 nm while some variation is observed when moving towards the edge of the sample. The maximum wavelength shift compared to that of the center is +/- 8 nm in the area of interest (corresponding to the patterned area of the pixelated silicon substrate.*

*Table S1. List of parameters used for the calculation of the transition energy e_1_-hh_1_ of the InGaN quantum wells following the parameters recommended by Vurgaftman and Meyer^1^.*

| Parameter | GaN | InN |  |
| --- | --- | --- | --- |
| *a* (Å) | 3.189 | 3.545 | *in-plane lattice parameter* |
| *E_g_* (eV) | 3.51 | 0.78 | *bandgap at 0K* |
| *α* (meV/K) | 0.909 | 0.245 | *Varshni coefficients* |
| *β* (K) | 830 | 624 |  |
| *b* (eV) | 1.4 | | *InGaN bandgap bowing parameter* |
| *c_13_* (GPa) | 106 | 92 | *elastic coefficients* |
| *c_33_* (GPa) | 398 | 224 |  |
| *D_1_* (eV) | -3.7 | -3.7 | *deformation potentials* |
| *D_2_* (eV) | 4.5 | 4.5 |  |
| *D_3_* (eV) | 8.2 | 8.2 |  |
| *D_4_* (eV) | -4.1 | -4.1 |  |
| *a_1_* (eV) | -4.9 | -3.5 |  |
| *a_2_* (eV) | -11.3 | -3.5 |  |
| *m_e_* | 0.2 | 0.07 | *electron effective mass* |
| *A_1_* | -7.21 | -8.21 | *Luttinger parameters* |
| *A_3_* | 6.68 | 7.57 |  |
| *ε* | 9.5 | 15.3 | *relative dielectric constant* |
| *Ry* (meV) | 25 | 6 | *bulk exciton binding energy* |
| *P_sp_* (C/m2) | -0.034 | -0.042 | *spontaneous polarization* |
| *d_13_* (pm/V) | -1.6 | -3.5 | *piezoelectric coefficients* |
| *d_33_* (pm/V) | 3.1 | 7.6 |  |


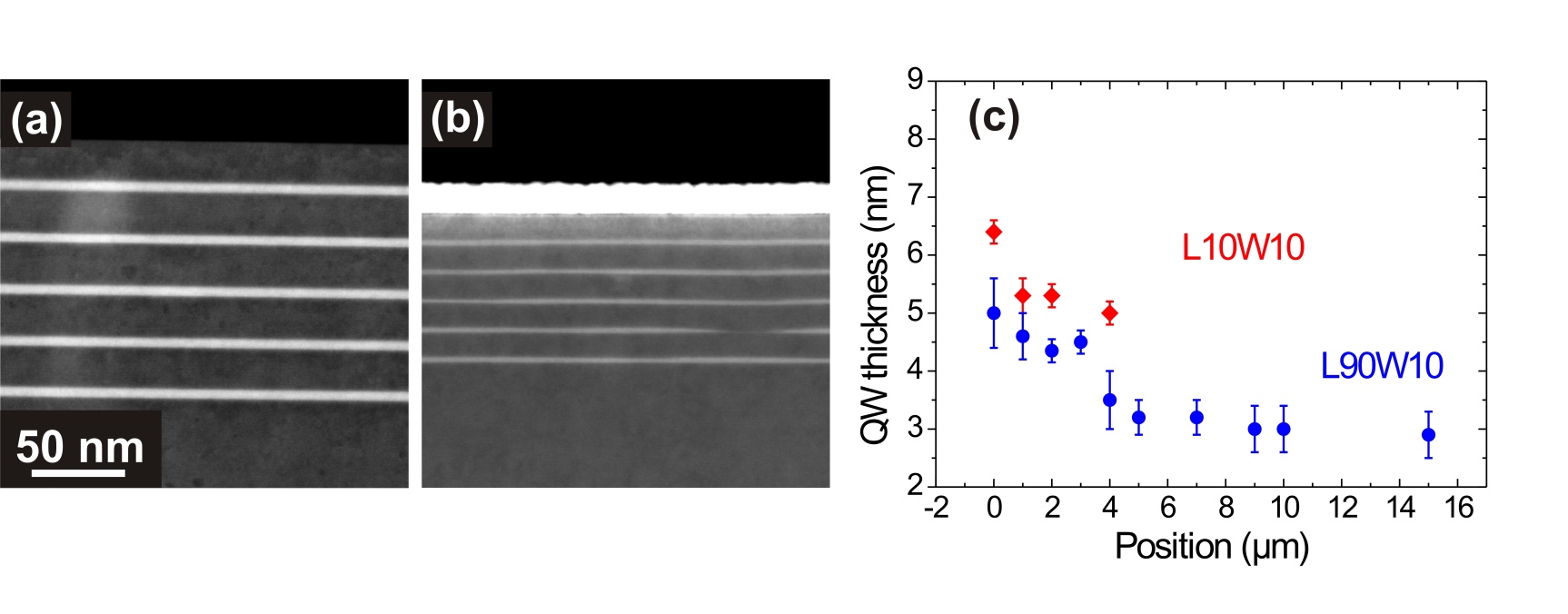


***Figure S2.*** *(a) Cross section HAADF STEM image of a mesa with a size of 10 µm and a trench width of 10 µm (L10W10) taken at 4 µm from the mesa edge. (b) Variation of the InGaN quantum well thickness as a function of the position (0 corresponds to the mesa edge) for 2 different mesas L10W10 and L20W10. The trench width is 10 µm for both mesas, the mesa size is 10 µm and 20 µm. A strong increase of the quantum well thickness at the mesa center is observed.*

**References**

1. Vurgaftman, I. & Meyer, J. R. Band parameters for nitrogen-containing semiconductors. *J. Appl. Phys.* **94**, 3675 (2003).

1. Corresponding author : bd@crhea.cnrs.fr [↑](#footnote-ref-1)
